# Intestine Bacterial Community Composition of Shrimp Varies Under Low- and High-Salinity Culture Conditions

**DOI:** 10.3389/fmicb.2020.589164

**Published:** 2020-11-16

**Authors:** Dongwei Hou, Renjun Zhou, Shenzheng Zeng, Dongdong Wei, Xisha Deng, Chengguang Xing, Lingfei Yu, Zhixuan Deng, Hao Wang, Shaoping Weng, Jianguo He, Zhijian Huang

**Affiliations:** ^1^State Key Laboratory of Biocontrol, Southern Marine Sciences and Engineering Guangdong Laboratory (Zhuhai), School of Marine Sciences, School of Life Sciences, Sun Yat-sen University, Guangzhou, China; ^2^Institute of Aquatic Economic Animals and Guangdong Province Key Laboratory for Aquatic Economic Animals, Sun Yat-sen University, Guangzhou, China

**Keywords:** intestine microorganism, microbial assembly, shrimp, salinity, disease

## Abstract

Intestine microbiota is tightly associated with host health status. Increasing studies have focused on assessing how host intestine microbiota is affected by biotic factors but ignored abiotic factors. Here, we aimed to understand the effects of salinity on shrimp intestine microbiota, by comparing the differences of intestine bacterial signatures of shrimp under low-salinity (LS) and high-salinity (HS) culture conditions. Our results found that intestine core bacterial taxa of shrimp under LS and HS culture conditions were different and that under HS contained more opportunistic pathogen species. Notably, compared with LS culture conditions, opportunistic pathogens (e.g., *Vibrio* species) were enriched in shrimp intestine under HS. Network analysis revealed that shrimp under HS culture conditions exhibited less connected and lower competitive intestine bacterial interspecies interactions compared with LS. In addition, under HS culture conditions, several opportunistic pathogens were identified as keystone species of intestine bacterial network in shrimp. Furthermore, the ecological drift process played a more important role in the intestine bacterial assembly of shrimp under HS culture conditions than that under LS. These above traits regarding the intestine microbiota of shrimp under HS culture conditions might lead to host at a higher risk of disease. Collectively, this work aids our understanding of the effects of salinity on shrimp intestine microbiota and helps for shrimp culture.

## Introduction

Intestine microbiota has fundamental roles in maintaining host health status (Hooper and Gordon, 2001; Boulangé et al., 2016). In this regard, it is important to determine the microbial signature of host intestines and their influencing factors. For aquatic animals, numerous diseases are linked with the dysbiosis of host intestine microbiota (Li et al., 2017; Dai et al., 2020; Huang et al., 2020). Extensive studies have shown that the intestine microbiota of aquatic animals is strongly affected by diet composition, trophic level, and developmental stage (Rungrassamee et al., 2013; Yan et al., 2016; Xiong et al., 2017; Liu et al., 2018; Walburn et al., 2019). These studies have primarily focused on assessing how the intestine microbiota of aquatic animals is affected by biotic factors but ignored abiotic factors. So far, research into the effects of abiotic factors on the intestine microbiota of aquatic animals has just begun. Some studies have shown that the intestine microbiota of aquatic animals is significantly affected by the salinity, ammonia, and temperature of rearing water (Sullam et al., 2012; Cornejo-Granados et al., 2018; Huang et al., 2018). Aquatic animals live in the water habitat with environm conditions (e.g., salinity, temperature) are constantly experience changes, and whether these abiotic factors can influence on host intestine microbiota, causing further adverse effects on host health status, remains unknown.

Pacific white shrimp (*Litopenaeus vannamei*), as a euryhaline specie, is present in a wide range of aquatic habitats and has become one of the most profitable aquaculture species in the word (FAO, 2016). However, the frequent occurrence of shrimp bacterial diseases, such as early mortality syndrome (EMS), acute hepatopancreatic necrosis disease (AHPND), hepatopancreas necrosis syndrome (HPNS), and white feces syndrome (WFS), has led to enormous economic losses every year worldwide (Sriurairatana et al., 2014; Lee et al., 2015; Choi et al., 2016; Huang et al., 2016; Xiong et al., 2017). In fact, the occurrence of *L*. *vannamei* bacterial diseases is closely associated with the obvious shifts in host intestine microbiota (Zhu et al., 2016; Hou et al., 2018; Huang et al., 2020; Zeng et al., 2020). Salinity is a very important abiotic factor affecting the intestine bacterial signatures (Zhang et al., 2016) and health status (Ponce-Palafox et al., 1997) of *L*. *vannamei*. It has been proposed that shrimp culturing under low salinity conditions is one way to counteracting disease problems and increasing production (Valencia-Castañeda et al., 2018). Accordingly, it is essential to explore what signatures of *L*. *vannamei* intestine microbiota are affected by salinity and whether this effect is related to the occurrence of the host disease.

The present study aims to explore the differences of the bacterial signatures in shrimp intestine under relative lower salinity (LS) and relative higher salinity (HS) culture conditions with the following questions: (i) What are the differences of the bacterial signatures in shrimp intestine under LS and HS culture conditions? (ii) What ecological processes shape the bacterial assembly of shrimp intestine under LS and HS culture conditions? We also provide the first attempt to elucidate the relationship between intestine bacterial signatures of shrimp under LS and HS culture conditions and the risk of host disease outbreaks. Our findings could provide a reference for the study of abiotic factors affecting the intestine microbiota of aquatic animals and help us establish the healthy culture strategies for shrimp.

## Materials and Methods

### Sample Collection

Two hundred seven shrimps were collected from Guangdong, Hainan, Guangxi, and Fujian provinces in China. The sampled shrimp culture ponds were of similar size (∼3,300 m^2^), water depth (∼1.5 m), and shrimp stocking density (the culturing began with a stocking of ∼200,000 shrimp larvae in each pond) (Supplementary Table S1). All shrimps were 60 days old, and the body average length was 10 cm. The salinity of rearing water (measured on site using a YSI handheld multiparameter instrument, Model YSI 380, YSI Incorporated, OH, United States) corresponding to 120 and 87 (from 40 and 29 culture ponds, respectively) shrimps is in 0‰ < salinity ≤ 5‰ and 5‰ < salinity < 10‰ groups (corresponding to LS and HS groups, according to Hou et al. (2017); Supplementary Table S1). Each shrimp intestine sample was aseptically dissected and placed in a 2-mL sterile centrifuge tube containing PBS. All shrimp intestine samples were stored at −80°C until DNA extraction.

### DNA Extraction, PCR Amplification, and Illumina MiSeq Sequencing

Shrimp intestine genomic DNA was extracted by a PowerFecal DNA Isolation Kit (Mobio, Carlsbad, CA, United States) following the manufacturer’s instruction. The V3–V4 regions of the bacterial 16S rRNA gene were amplified using the primers 338F and 806R. PCR was performed in 50-μL reactions, with each containing 50 ng of purified DNA as a template, and the following thermocycling conditions were used: 25 cycles of denaturation at 95°C for 30 s, annealing at 55°C for 30 s, and extension at 72°C for 45 s, with a final elongation at 72°C for 10 min. Each sample was pooled and purified using a PCR fragment purification kit (Qiagen, GmbH, Hilden, Germany). Equimolar amounts of amplicons from each sample were pooled and then sequenced using a MiSeq 2 × 300 bp platform (Illumina, San Diego, CA, United States) by Majorbio Bio-Pharm Technology Co., Ltd. (Shanghai, China). The original MiSeq 16S rRNA sequence data supporting the findings of this study have been deposited in the NCBI BioProject database under the accession number PRJNA545396.

The paired-end sequences were merged using FLASH (Magoč and Salzberg, 2011) and then processed following the quantitative insights into microbial ecology pipeline (QIIME version 1.9.0) (Caporaso et al., 2010). In short, sequences with ambiguous bases or truncations at any site for more than three consecutive bases and receiving a Phred quality score (Q) <20 were removed. Subsequently, chimeric sequences were removed using the UCHIME algorithm (Edgar et al., 2011). The bacterial phylotypes were identified using UCLUST (Edgar, 2010) and classified into operational taxonomic units (OTUs) at a 97% cutoff. The most abundant sequence from each OTU was selected as a representative and was taxonomically assigned to a closed reference genome using the RDP Classifier algorithm^1^ (Silva SSU database 128), enabling a close relative to be identified for each OTU. The α-diversity estimates were calculated by analyzing the observed species using QIIME (Version 1.9.0). Core taxa provide information on microorganisms was putatively identified important in the host intestine, and core taxa were identified via the following criteria: core OTUs in the LS and HS groups were those present in ≥90% (Qin et al., 2010; Ainsworth et al., 2015) of 120 or 87 intestine samples, respectively.

### Ecological Process Analysis

We used the mean nearest taxon distance (MNTD) measure to determine which processes govern the shrimp intestine bacterial assembly. To evaluate the degree of non-random phylogenetic relatedness, the “standardized effect size” of the phylogenetic community structure (ses.MNTD) was calculated for MNTD by determining the difference between phylogenetic distances in the observed communities vs. those measured for the null communities (999 randomizations), which was divided by the standard deviation of the phylogenetic distances in distribution (Kembel et al., 2010). These analyses were implemented in the R (Ver 3.3.2) environment using the package “Picante” (R Core Team, 2015). Similarly, the mean distance between each taxon and its nearest neighbor (β-MNTD) between a given pair of samples was computed by random shuffling of OTUs and their abundances across phylogenetic tips, which reflects the dissimilarity between the bacterial communities (Stegen et al., 2013). The difference between the observed β-MNTD and the mean of the null distribution is referred to as the β-NTI. The fractions of all β-NTI values that were > 2 or < −2 denote the relative influences of heterogeneous and homogeneous selection, respectively (Vellend, 2010). The β-NTI values in combination with the Bray–Curtis distance (based on the Raup–Crick distance, RC_Bray_) was further used to quantify the contributions of major ecological processes that determine bacterial assembly in the shrimp intestine. Both of the β-NTI and RC_Bray_ values were used to estimate the contributions of homogenizing dispersal and dispersal limitation. That is, the fractions of pairwise comparisons with |β-NTI| < 2 but RC_Bray_ > 0.95 or < −0.95 were used to estimate the relative importance of dispersal limitation or homogenizing dispersal processes, respectively (Stegen et al., 2013). The fraction of pairwise comparisons with |β-NTI| < 2 and |RC_Bray_| < 0.95 represented the component of compositional turnover governed by the ecological drift process (Stegen et al., 2015). Among these processes, selection or ecological drift is unambiguously deterministic or stochastic (Chase et al., 2011; Vellend et al., 2014), whereas dispersal can be either deterministic, stochastic, or both (Hanson et al., 2012).

### Statistical Analysis

Partial least squares discrimination analysis (PLS-DA) was performed to assess the bacterial similarity based on the Bray–Curtis distance. Then, the relationships between bacterial OTUs in the LS and HS groups were studied using Venn analysis. Welch’s *t*-test was used to compare the bacterial diversity indices and differentially abundant taxa (at OTU or genus level) between the two groups, and then the Sidak was used for multiple test correction. Further, we evaluated the extent of bacterial interspecies interactions of shrimp intestine in the two groups using an open-accessible pipeline^2^ (Deng et al., 2012). To quantify the interspecies interactions, a set of topological properties were calculated, including the average path length, clustering coefficient, and co-occurrences (Mej, 2003), and the resulting network was visualized via Cytoscape 3.6.1.

## Results

### Bacterial Diversity of Shrimp Intestine in the LS and HS Groups

**FIGURE 1:**
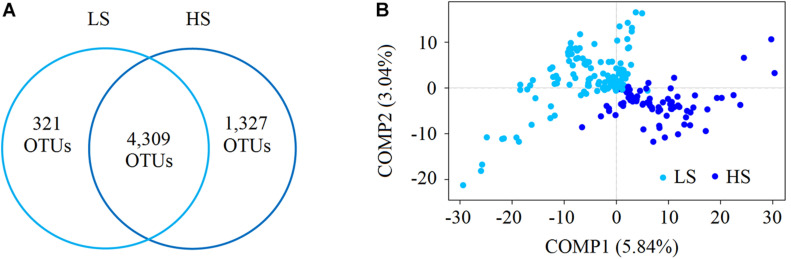
Compare the bacterial diversity of shrimp intestine in the LS and HS groups. **(A)** Schematic drawing showing the detected shrimp intestine bacterial OTUs found in the LS and HS groups using the Venn analysis. There were 4,309 OTUs shared between the two groups, while 321 and 1,327 OTUs were only in the LS and HS groups, respectively. **(B)** The PLS-DA of the bacterial community structure of shrimp intestine in the LS and HS groups based on the Bray–Curtis distance, and the results showed marked differences in the two groups.

We recovered 9,932,526 high-quality sequences, and after subsampling 29,499 sequences per sample, 6,106,293 sequences were retained (Supplementary Table S2). A total of 5,957 intestine bacterial OTUs of shrimp were identified in this study, among which 4,630 and 5,636 OTUs were identified in the LS and HS groups, respectively (Figure 1A). Among these OTUs, 4,309 were shared between the two groups, while 321 and 1,327 were only in the LS and HS groups, respectively (Figure 1A), revealing that the OTU number in the two groups exhibited a high level of variation. Then, the α-diversity indices were calculated using the rarefaction curves at the OTU level at a sequencing depth of 29,499 with 1,000 iterations, where Shannon, Simpson, Ace, and Chao indices were stable (Supplementary Figure S1). These α-diversity indices in the LS group were slightly higher than in the HS group, but Welch’s *t*-test results indicated that there was no significant (*P* > 0.05) difference between the two compared groups (Supplementary Table S3). For the β-diversity, the PLS-DA results showed a marked variation in the shrimp intestine bacterial structures in the two groups (Figure 1B).

### Core Bacterial Taxa of Shrimp Intestine in the LS and HS Groups

**FIGURE 2:**
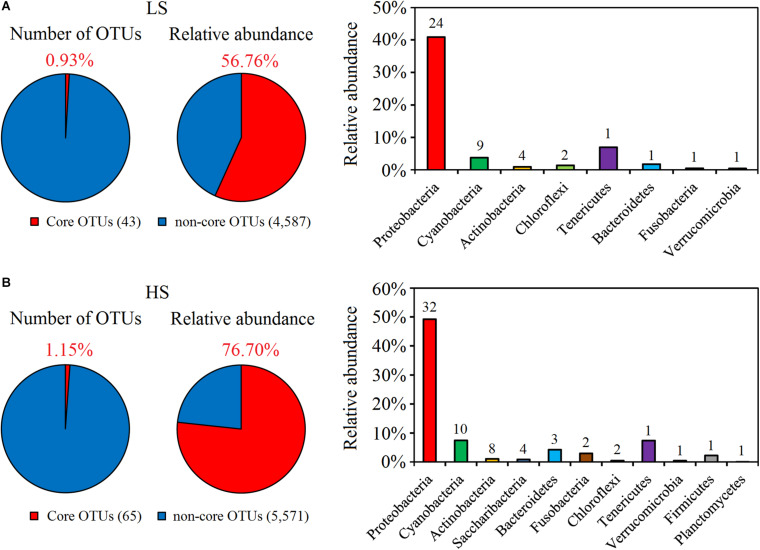
Abundance and composition of core OTUs of shrimp intestine in the LS and HS groups. **(A)** Forty-three OTUs were identified as the core OTUs in the LS group, accounting for 56.76% of all intestine bacterial sequences obtained, which belonged to Proteobacteria, Cyanobacteria, Actinobacteria, Chloroflexi, Tenericutes, Bacteroidetes, Fusobacteria, and Verrucomicrobia. **(B)** Sixty-five OTUs were identified as the core OTUs in the HS group, accounting for 76.70% of all intestine bacterial sequences obtained, which belonged to Proteobacteria, Cyanobacteria, Actinobacteria, Saccharibacteria, Bacteroidetes, Fusobacteria, Chloroflexi, Tenericutes, Firmicutes, Verrucomicrobia, and Planctomycetes.

The core taxa of shrimp intestine bacterial communities in the two groups were identified based on the frequency of OTU occurrence. Forty-three and 65 OTUs were identified as the core OTUs in the LS and HS groups, accounting for 56.76 and 76.70% of all intestine bacterial sequences obtained, respectively (Figure 2). At the phylum level, the intestine core bacterial OTUs of shrimp in the LS group were Proteobacteria, Cyanobacteria, Actinobacteria, Chloroflexi, Tenericutes, Bacteroidetes, Fusobacteria, and Verrucomicrobia (Figure 2A), while those observed for the HS group included Proteobacteria, Cyanobacteria, Actinobacteria, Saccharibacteria, Bacteroidetes, Fusobacteria, Chloroflexi, Tenericutes, Firmicutes, Verrucomicrobia, and Planctomycetes (Figure 2B). Thus, the intestine core taxa of shrimp in the two groups were distinct from each other. In addition, *Vibrio* OTU4739 and *Vibrio* OTU5086 were identified as core OTUs in the LS group, while *Vibrio* OTU5086, *Vibrio* OTU4739, *Candidatus* Bacilloplasma OTU725, *Vibrio* OTU5173, *Vibrio* OTU69, *Vibrio* OTU5511, *Vibrio* OTU1341, *Vibrio* OTU688, and *Vibrio* OTU815 were identified as core OTUs in the HS group (Supplementary Table S4), showing that the core taxa under HS culture conditions included more opportunistic pathogen species.

### Opportunistic Pathogens Enriched in Shrimp Intestine in the HS Group

**FIGURE 3:**
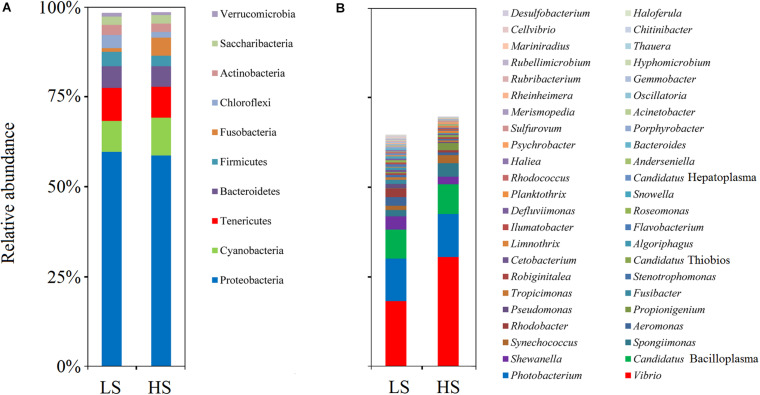
Bacterial profiles of shrimp intestine in the LS and HS groups. Relative abundance of phyla **(A)** and genera **(B)** in the LS and HS groups. Proteobacteria, Cyanobacteria, Tenericutes, Bacteroidetes, Firmicutes, Fusobacteria, Chloroflexi, Actinobacteria, Saccharibacteria, and Verrucomicrobia were the 10 most abundant phyla in shrimp intestine, while *Vibrio*, *Photobacterium*, *Candidatus* Bacilloplasma, *Shewanella*, *Spongiimonas*, *Synechococcus*, *Aeromonas*, *Rhodobacter*, *Propionigenium*, and *Pseudomonas* were the 10 most abundant genera, with the relative abundances of these genera varying between the two groups.

**FIGURE 4:**
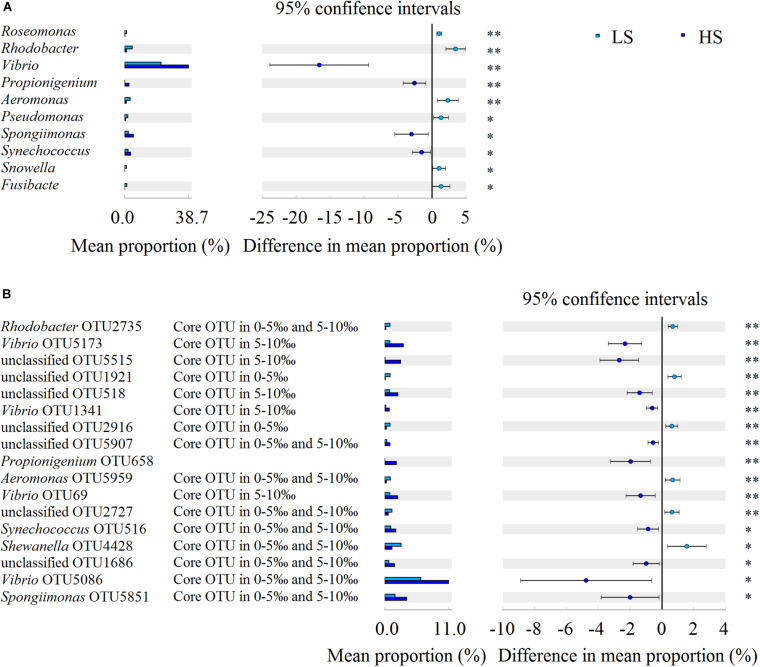
Bacterial taxonomic comparison of shrimp intestine in the LS and HS groups. Welch’s *t*-test results showed that the relative abundances of the 10 genera **(A)** and 17 OTUs **(B)** had significant differences between the LS and HS groups (^∗^*P <* 0.05, ^∗∗^*P <* 0.01).

To compare the intestine bacterial taxonomic compositions of shrimp in the two groups, we assessed their bacterial profiles at the phylum and genus levels. At the phylum level, Proteobacteria, Cyanobacteria, Tenericutes, Bacteroidetes, Firmicutes, Fusobacteria, Chloroflexi, Actinobacteria, Saccharibacteria, and Verrucomicrobia were the 10 most abundant phyla in shrimp intestine (Figure 3A). At the genus level, *Vibrio*, *Photobacterium*, *Candidatus* Bacilloplasma, *Shewanella*, *Spongiimonas*, *Synechococcus*, *Aeromonas*, *Rhodobacter*, *Propionigenium*, and *Pseudomonas* were the 10 most abundant genera in shrimp intestine, with the relative abundances of these genera varying between the two groups (Figure 3B). Welch’s *t*-test results further showed that the relative abundances of the 10 genera and 17 OTUs were significant differences (*P* < 0.05 in all cases) between the two groups. The relative abundances of *Vibrio*, *Propionigenium*, *Spongiimonas*, and *Synechococcus* were overrepresented in the HS group, whereas those of *Roseomonas*, *Rhodobacter*, *Aeromonas*, *Pseudomonas*, *Snowella*, and *Fusibacter* were higher in the LS group (Figure 4A). At the OTU level, the relative abundances of *Vibrio* OTU5173, unclassified OTU5515, unclassified OTU518, *Vibrio* OTU1341, unclassified OTU5907, *Propionigenium* OTU658, *Vibrio* OTU69, *Synechococcus* OTU516, unclassified OTU1686, *Vibrio* OTU5086, and *Spongiimonas* OTU5851 were overrepresented in the HS group. In contrast, the relative abundances of *Rhodobacter* OTU2735, unclassified OTU1921, unclassified OTU2916, *Aeromonas* OTU5959, unclassified OTU2727, and *Shewanella* OTU4428 were higher in the LS group (Figure 4B). Interestingly, *Vibrio* OTU5173, *Vibrio* OTU1341, *Vibrio* OTU69, and *Vibrio* OTU5086 were core OTUs and overrepresented in the HS group (Figure 4B).

### Opportunistic Pathogens Are Keystone Species of Shrimp Intestine Interspecies Interactions in the HS Group

**FIGURE 5:**
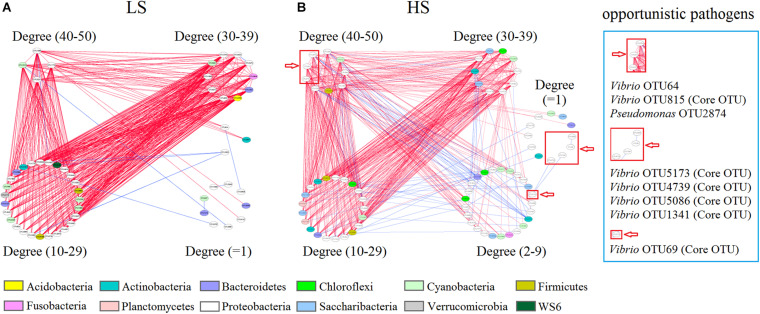
Bacterial co-association network of shrimp intestine in the LS and HS groups. Shrimp intestine bacterial interspecies interactions in the LS **(A)** and HS **(B)** groups. Each node represents a bacterial OTU. The colors of nodes indicate OTUs affiliated to different phyla. A *blue edge* indicates a positive interaction between two individual nodes, whereas a *red edge* indicates a negative interaction.

To investigate whether salinity significantly affected the intestine bacterial co-association networks of shrimp, the OTU table was split into two datasets (the bacterial OTUs in the two groups) to quantify the interspecies interactions. Network analysis results suggested that the average degree indices of shrimp intestine bacterial communities were 17.40 and 16.34, while the average clustering coefficient index values of 0.58 and 0.59 but graph density index values of 0.34 and 0.22 were observed in the LS and HS groups, respectively (Supplementary Table S5), revealing that the shrimp intestine bacterial network was more complex and better connected in the LS group. Moreover, the observed co-associations were predominantly negative, and the relative negative co-occurrences were 97.40 and 89.35% in the LS and HS groups, respectively (Figure 5), indicating obviously higher interspecies competitive activities of the shrimp intestine bacterial community in the LS group.

Additionally, *Vibrio* OTU64, *Vibrio* OTU815, and *Pseudomonas* OTU2874 (degree = 49, 44, and 41, respectively) were keystone species (higher degree nodes were reported as keystone species) that had numerous neighbors in the HS group, while *Vibrio* OTU5173, *Vibrio* OTU4739, *Vibrio* OTU5086, *Vibrio* OTU1341, and *Vibrio* OTU69 (degree = 2, 1, 1, 1, and 1) had only a few neighbors in the HS group network (Figure 5B). Interestingly, *Vibrio* OTU815 (a keystone species), *Vibrio* OTU5173, *Vibrio* OTU4739, *Vibrio* OTU1341, *Vibrio* OTU69, and *Vibrio* OTU5086 were also identified as shrimp intestine core OTUs in the HS group (Figure 5B and Supplementary Table S6), with *Vibrio* OTU5173, *Vibrio* OTU1341, *Vibrio* OTU69, and *Vibrio* OTU5086 being overrepresented in this group (Figure 4B). Further, most of the neighbors in the bacterial co-association network negatively interacted with *Vibrio* OTU64, *Vibrio* OTU815, and *Pseudomonas* OTU2874 from shrimp intestine in the HS group (Figure 5B). Thus, in the HS group, some opportunistic pathogens (especially *Vibrio* OTU815) are keystone species involved in bacterial interspecies interactions of shrimp intestine.

### Ecological Processes Governing the Shrimp Intestine Bacterial Assembly

**FIGURE 6:**
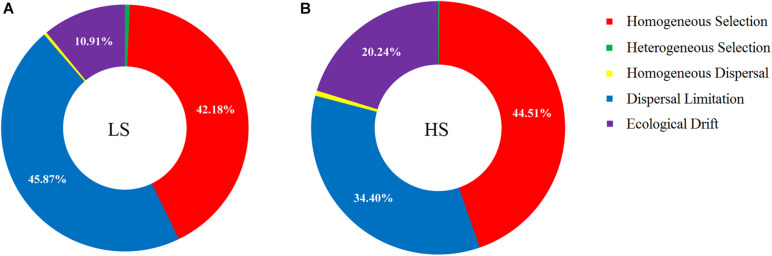
Ecological process analyses on shrimp intestine bacterial assembly in the LS and HS groups. **(A)** Approximately half of the observed variation was attributed to dispersal limitation and homogeneous selection processes in the LS group, while the drift process contributed to only 10.91% variation. **(B)** Over one-third of the variation was attributed to homogeneous selection and dispersal limitation processes in the HS group, while the ecological drift process contributed to 20.24% of the observed variation.

The relative contributions of major ecological processes were quantified to evaluate the shrimp intestine bacterial assembly in the two groups. The results showed that approximately half of the observed variation was attributable to dispersal limitation (45.87%) and homogeneous selection (42.18%) processes in the LS group, while the drift process contributed to only 10.91% variation (Figure 6A). In the HS group, over one-third of the variation was attributed to homogeneous selection (44.51%) and dispersal limitation (34.40%) processes, while the ecological drift process contributed to 20.24% of the observed variation (Figure 6B). By contrast, the contributions of homogenizing dispersal and heterogeneous selection processes were much less pronounced for shrimp intestine bacterial communities in the two groups (Figure 6). Importantly, these findings indicated that the relative contribution of ecological drift processes that govern the shrimp intestine bacterial community in the HS group was higher than in the LS group.

## Discussion

The intestine microbiota of shrimp is increasingly recognized to facilitate host health, and its influencing factors have been extensively studied. To the best of our knowledge, the intestine microbiota of shrimp is not only affected by biotic factors (Burns et al., 2016; Dai et al., 2017; Zeng et al., 2017) but also by abiotic factors (e.g., salinity and temperature) (Zhang et al., 2016; Huang et al., 2018). Our study reinforced the evidence that the shrimp intestine bacterial communities are influenced by the salinity of rearing water, with marked variations observed of taxonomic composition, core taxa, and interspecies interactions under different salinity culture conditions. Similarly, there was a study found that the most important biological factor in structuring the shrimp intestine microbiota was the marine and freshwater environment, and the freshwater showed higher bacterial diversity than marine shrimps (Cornejo-Granados et al., 2018). In addition, other studies also indicated that strong influences of salinity on the taxonomic composition and interspecies interactions of intestinal microbiota in other aquatic animals, such as fish and crayfish (Zhang et al., 2016; Liu et al., 2020). Thus, salinity affects the intestine microbiota of a wide variety of aquatic animals. In addition, we identified the indicative bacterial taxa of shrimp intestine under LS and HS culture conditions and found that the relative abundances of 17 bacterial OTUs in shrimp intestine were significantly different under LS and HS culture conditions. Especially, some opportunistic pathogens (especially some *Vibrio* species) were enriched in shrimp intestine under HS culture conditions. It is worth noting that these opportunistic pathogens identified as being enriched in host intestine were generally accompanied by shrimp bacterial disease outbreaks, as shown in previous works by our lab and others (Xiong et al., 2017; Huang et al., 2020).

Based on the recent progress, it is apparent that the specific microbes present in intestines are selected for by hosts (Burns et al., 2016). Depending on the environment in which the host lives, the specific microbes selected by the host intestine will be different (Mortzfeld et al., 2016). Thus, our study raises an important question: what specific microbes are being selected for in shrimp intestine under different salinity conditions? To address this concern, we compared the intestine core bacterial taxa compositions of shrimp cultured under LS and HS conditions and found that different core bacterial taxa were selected by shrimp intestine. For the host, intestine core microbial taxa may be acquired early in life, and because they make substantial contributions to basic intestine microbial functions (Shade and Handelsman, 2012), they may be actively retained and managed by hosts (Franzosa et al., 2015). Generally, intestine core microbial taxa are considered beneficial to the host health (Walter and Ley, 2011). However, our study found that the intestine core taxa of shrimp under HS culture conditions contained more opportunistic pathogen species. A possible reason may explain such observations: to improve host fitness, hosts must recruit suitable microbial taxa that perform a variety of functions (McFall-Ngai et al., 2013), and the colonization of bacteria in intestines from external environments occurs as a result of deterministic processes (Xiong et al., 2018), but stochastic processes also promote the establishment and success of external microbial taxa, including both symbionts and opportunistic pathogens (Mallon et al., 2015). A previous research has found that the increased importance of the ecological drift process in the shrimp intestine microbiota promotes the acquisition of opportunistic pathogens (Zhu et al., 2016). In our study, a much greater contribution of the ecological drift process governing the shrimp intestine bacterial assembly was also observed under HS than LS culture conditions, potentially explaining why so many opportunistic pathogen species were part of the core taxa and enriched in shrimp intestine under HS culture conditions.

Additionally, the complexity of microbial interspecies interactions in host intestine is closely associated with community functional potential (Riva et al., 2017). More importantly, the co-occurrence equilibrium of intestine microbiota may provide an index for evaluating the risk of host disease (Xiong et al., 2018). In this study, intestine bacterial interspecies interactions of shrimp under LS culture conditions were more complex and better connected than those observed under HS culture conditions. In particular, under HS culture conditions, some opportunistic pathogens were keystone species and played important roles in intestine bacterial interspecies interactions of shrimp. These traits suggested to some extent that shrimp cultured under HS culture conditions may be at high risk of disease. Moreover, we observed that most of all the neighbors of bacterial interspecies interactions in shrimp intestine negatively interacted with opportunistic pathogens under HS culture conditions. A potential reason for this observation is that the resistance to pathogen colonization is mediated by multiple microbial taxa that interact in a context-dependent manner (Schubert et al., 2015). However, in the host intestine, the opportunistic pathogens can create ecological niches that facilitate their expansion and advantages to outcompete commensals (Thiennimitr et al., 2011; Mallon et al., 2015). These abilities may explain why some opportunistic pathogens were keystone species of intestine bacterial interspecies interactions of shrimp under HS culture conditions.

## Conclusion

This study aimed to understand what signatures of shrimp intestine microbiota are affected by salinity and whether this effect is related to risk of host disease outbreaks, by comparing the intestine microbiota of shrimp under LS and HS culture conditions. Our findings illustrated that intestine core bacterial taxa of shrimp under HS culture conditions contained more opportunistic pathogen species and that some of them were enriched. The potential reason was that the ecological drift process plays a more important role in the intestine bacterial assembly of shrimp under HS culture conditions than under LS culture conditions. In addition, under HS culture conditions, several opportunistic pathogens were keystone species of bacterial interspecies interactions in shrimp intestine. These results suggested that shrimp under HS culture conditions may be at a higher risk of disease outbreaks. Collectively, our study provides ecological insights for understanding the effects of salinity on shrimp intestine microbiota and contributes to the establishment of healthy shrimp culture strategies.

## Data Availability Statement

The datasets generated for this study can be found in the online repositories. The names of the repository/repositories and accession number(s) can be found in the article/ Supplementary Material.

## Author Contributions

DH, RZ, SZ, DW, XD, CX, LY, ZD, and HW collected the samples and performed the experiments. DH, RZ, and SZ analyzed the data. DH and ZH wrote the manuscript. DH, ZH, SW, and JH contributed to the conception of the work. ZH was primarily responsible for the final content. All authors contributed to the article and approved the submitted version.

## Conflict of Interest

The authors declare that the research was conducted in the absence of any commercial or financial relationships that could be construed as a potential conflict of interest.

## Footnotes

1 http://rdp.cme.msu.edu/

2 http://ieg2.ou.edu/MENA

## Supplementary Material

Supplementary Figure S1 The α-diversity rarefaction curves for all shrimp intestine samples. All curves were calculated at the maximum depth of 29,499 reads per sample. **(A)** Shannon, **(B)** Simpson, **(C)** Ace, and **(D)** Chao indices.

Supplementary Table S1 Sampling sites characteristics.

Supplementary Table S2 Summary of sequence numbers and bacterial OTUs for shrimp intestine.

Supplementary Table S3 Bacterial α-diversity indices of shrimp intestine and statistical testing of these indices by the Welch’s *t*-test in the LS and HS groups.

Supplementary Table S4 Intestine core OTUs of shrimp in the LS and HS groups. The red word indicates that the species are core taxa both in two groups. And the highlighted in gray indicates that the species are opportunistic pathogens in shrimp culture.

Supplementary Table S5 Topological properties of MENs on shrimp intestine bacterial communities in the LS and HS groups.

Supplementary Table S6 Network nodes’ centrality indices of shrimp intestine bacterial communities in the LS and HS groups.
