# Supplementary figures and images for: Intestine Bacterial Community Composition of Shrimp Varies Under Low- and High-Salinity Culture Conditions
Source: Front Microbiol. 2020 Nov 16;11:589164. doi: 10.3389/fmicb.2020.589164 (PMC7701045; doi:10.3389/fmicb.2020.589164)

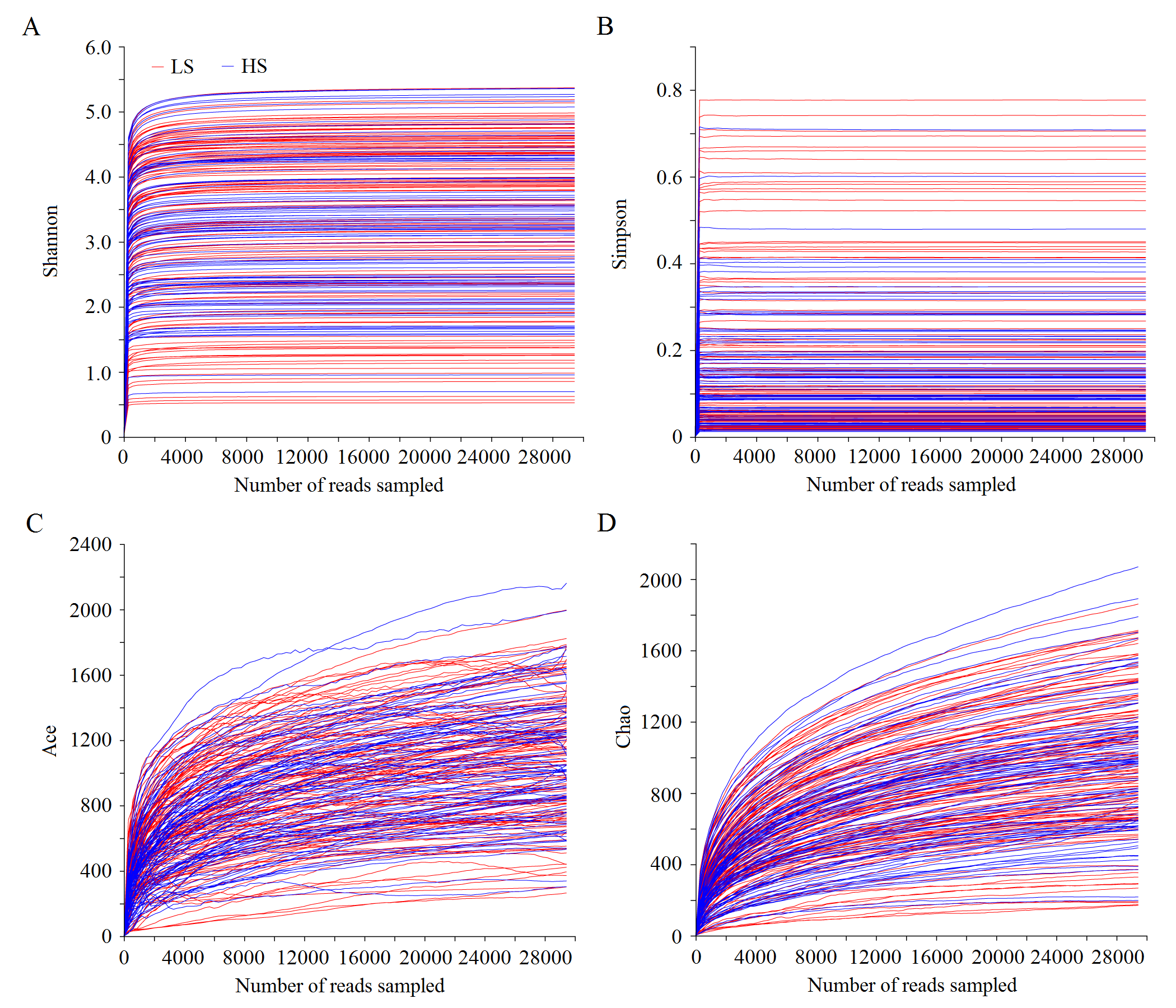

Supplement: Supplementary Figure S1 — The α-diversity rarefaction curves for all shrimp intestine samples. All curves were calculated at the maximum depth of 29,499 reads per sample. (A) Shannon, (B) Simpson, (C) Ace, and (D) Chao indices. [file Image_1.TIF]
